# Case report of successful management of intraoperative tracheal rupture during thoracoscopic esophageal resection in patient with esophageal cancer

**DOI:** 10.1016/j.ijscr.2019.02.045

**Published:** 2019-03-09

**Authors:** Аlexander Khitaryan, Ismail Miziev, Camil Veliev, Olga Voronova, Anastasiya Golovina, Raisa Zavgorodnyaya, Sergey Kovalev, Albert Alibekov, Аlexey Orekhov

**Affiliations:** a NGHCI Railway Clinical Hospital at the “Rostov-Glavnyy” Station, OAO Russian Railways, Varfolomeeva Street 92, Rostov-on-Don, Russian Federation; b FSBEI HE Rostov State Medical University of the Ministry of Health of the Russian Federation, Nakhichevansky Lane 19, Rostov-on-Don, Russian Federation; c FSBEI HE Kabardino-Balkarian State University Named After Berbekov H.M., Chernyshevskiy Street 173, Nalchik, Russian Federation

**Keywords:** Esophageal cancer, Thoracoscopic esophageal resection, Intraoperative complications, Trachea rupture, Case report

## Abstract

**Introduction:**

A rupture of the membranous part of trachea during thoracoscopic and transhiatal resection of esophagus is a rare complication that occurs in 0.4% of cases. This complication often requires thoracotomy and is associated with prolonged pulmonary ventilation, long-term pleural draining due to persistent air leakage and development of a tracheopleural fistula, prolonged hospitalization, and high risk of septic and secondary cardiorespiratory complications.

**Presentation of case:**

A 52-year-old woman complained of difficulty eating solid food, impaired swallowing, persistent pain behind the sternum, nausea, sickness and weight loss. EGD revealed tumor almost completely obturating the lumen in the middle third of the esophagus. The histopathological examination showed esophageal SCC. CT scan confirmed 33 × 29 × 55 mm tumor. The patient underwent thoracoscopic esophageal resection during which two defects of the tracheobronchial tree with sizes of 15 mm and 30 mm were detected and then successfully sewn using intracorporeal continuous Stratafix 3.0 suture. The patient was diagnosed with cancer of middle third of esophagus pT3N1M0, stage IIIB.

**Discussion:**

Thorough selection of patients undergoing thoracoscopic esophageal resection together with gentle manipulations with the esophagus in the area adjacent to the trachea can prevent intraoperative tracheobronchial damages. Timely diagnosis of such serious complications makes it possible to successfully manage them using thoracoscopic techniques.

**Conclusion:**

Intraoperative tracheal and bronchial ruptures can be successfully managed with thoracoscopic intracorporeal suture.

## Highlights

• Intraoperative ruptures of trachea during thoracoscopic esophageal resection are very serious complications.
• A 52-year-old woman had cancer of middle third of esophagus T3N1M0, stage IIIB.
• The woman underwent thoracoscopic esophageal resection with gastric tube plasty.
• Successful management of intraoperative tracheal and bronchial rupture with use of the intracorporeal suture.
• The patient had an uneventful, standard recovery and was discharged 12 days after the surgery.

## 1 Introduction

Esophageal cancer is the 19th most common cancer in the European Union [1] and its surgery is considered to be one of the most extensive and traumatic oncological procedures with high complication rates, varying from 40 to 80% and in-hospital mortality varying from an average of 7.5% to below 5% in experienced centers [2].

A rupture of the membranous part of trachea during thoracoscopic and transhiatal resection of esophagus is a rare complication that occurs in 0.4% of cases and more exclusively during open esophageal surgery (0–0.2% cases) [[3], [4], [5], [6]]. The intraoperative ruptures can be caused by dystrophic changes of the esophageal membrane of trachea and connective esophageal-tracheal fibers, incautious traction of the esophagus, especially in case of periesophageal inflammation. Also, a large size of an esophageal tumor and its invasion into adjacent organs can be reasons for tears. This complication often requires thoracotomy and is associated with prolonged pulmonary ventilation, long-term pleural draining due to persistent air leakage and development of a tracheopleural fistula, prolonged hospitalization, and high risk of septic and secondary cardiorespiratory complications.

In this case report our objective was to present the experience of thoracoscopic closure of the membranous part of the trachea defect, which occurred during thoracoscopic resection for esophageal cancer.

The operation was performed in the surgical department of the non-governmental clinical hospital.

The work has been reported in line with the SCARE criteria [7].

## 2 Presentation of case

A 52-year-old Caucasian woman was admitted to our surgical department. She complained of difficulty eating solid food, impaired swallowing, persistent nagging pain behind the sternum, periodic heartburn and nausea, sickness. The woman had lost 6 kg in the previous 2 months. She had been using antacid medications without prescription for a month with no effect. The patient denied smoking and alcohol intake. The woman had no previous surgical or another medical history.

The diagnostic measures included esophagogastroduodenoscopy (EGD) with biopsy and contrast chest spiral computed tomography (contrast CT) scan. EGD revealed tumor almost completely obturating the lumen in the middle third of the esophagus (25 cm from incisors), impassable for the endoscope. A biopsy was taken. The histopathological examination showed esophageal squamous cell carcinoma (SCC).

**Fig. 1:**
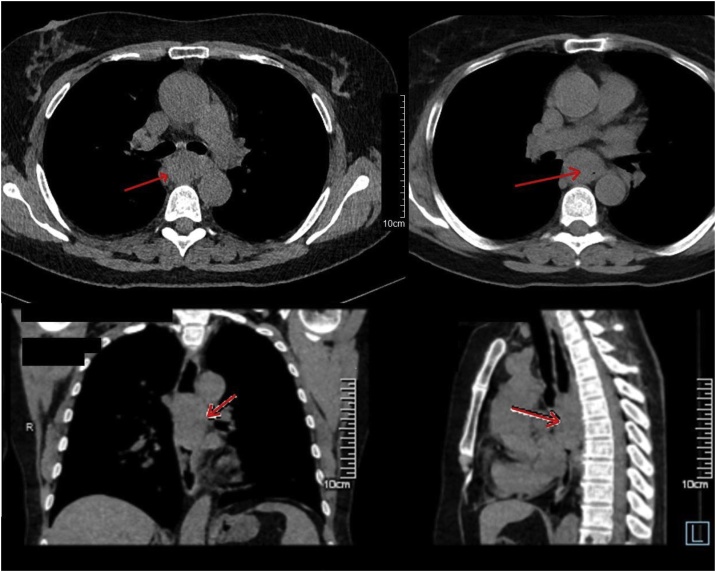
Preoperative contrast CT (see comments in the text).

CT scan confirmed the diagnosis, showing 33 × 29 × 55 mm tumor of the middle third of the esophagus intensively and irregularly absorbing contrast (Fig. 1). Above the tumor, the esophagus was dilatated and contained a little amount of fluid. There was one increased paratracheal lymph node in mediastinum with the size of 8 mm. No other pathologic changes were diagnosed. Endoscopic ultrasound (EUS) was not performed because of patient’s medical insurance peculiarities. The tracheobronchoscopy showed no invasion of the tumor into the tracheobronchial tree and no other pathological changes. Abdomen CT revealed no distant metastases and no abdominal lymph nodes involvement.

The patient was diagnosed with cancer of middle third of esophagus T3N1M0, stage IIIB. According to national clinical standards the patient underwent surgical treatment [8]. The time from the moment of admission to the hospital until the operative treatment was 3 days.

**Operative technique** (the operative technique of thoracoscopic stage is described in details in our previous case report [9]):

The patient was initially placed in the supine position, and a double lumen endotracheal tube was placed in preparation for the thoracoscopic mobilization of the esophagus. The abdominal stage was open (laparotomy). The gastric tube was formed by dissecting gastrocolic ligament along the greater curvature from the body of the stomach to the subcardial part preserving the right gastroepiploic artery. Then short gastric vessels, gastrophrenic ligament, abdominal part of the esophagus, hepatogastric ligament were cut. The left gastric artery was cut after lymph node dissection in the area of hepatic and splenic arteries, celiac trunk. The gastric tube was formed from the fundus by resecting small curvature by 2 cassettes of 100 mm linear stapler (Covidien, Ireland), leaving 35 mm gastric pouch. The phrenic vein together with a part of diaphragmatic cruses was cut, expanding the esophageal opening of the diaphragm for gastric conduit and the tumor withdrawal. Then the laparotomy incision was closed and the patient was turned to the prone position for the thoracoscopic mobilization of the esophagus and creation of the intrathoracic anastomosis.

Further four trocars were placed into the right pleural cavity: two 10 mm, one 12 mm and one 5 mm. After the right lung collapse, mediastinal pleura was incised from diaphragm continuing up to 2–4 cm above the carina using EnSeal tissue sealer. The azygos vein was clipped with Hem-o-lock (Weck Closure Systems, Research Triangle Park, NC, USA) and cut.

The esophagus was mobilized with the fat tissue and the lymph nodes around it up to the planes of the aorta, the pericardial sac, and contralateral pleura. Then the esophageal tumor with sizes of 6.5 × 4 cm tightly adjoined to the first main bronchus and trachea became visible. The tumor was hard to dissect in the plain of the trachea and the bronchus. The esophagus was mobilized up to the level of the pleural dome, taken on the holder and separated from the tracheobronchial tree.

**Fig. 2:**
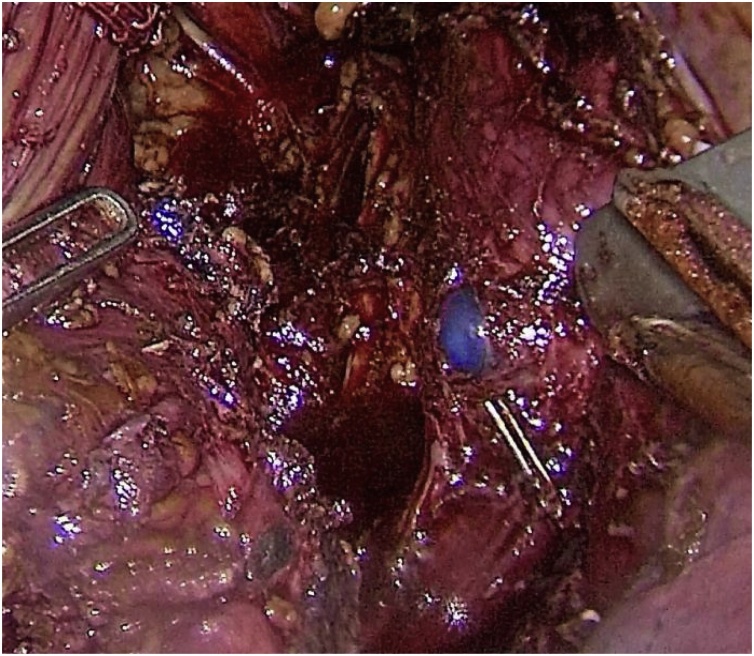
The double-lumen endotracheal tube blue bronchial cuff is seen in the right main bronchus through the rupture.

**Fig. 3:**
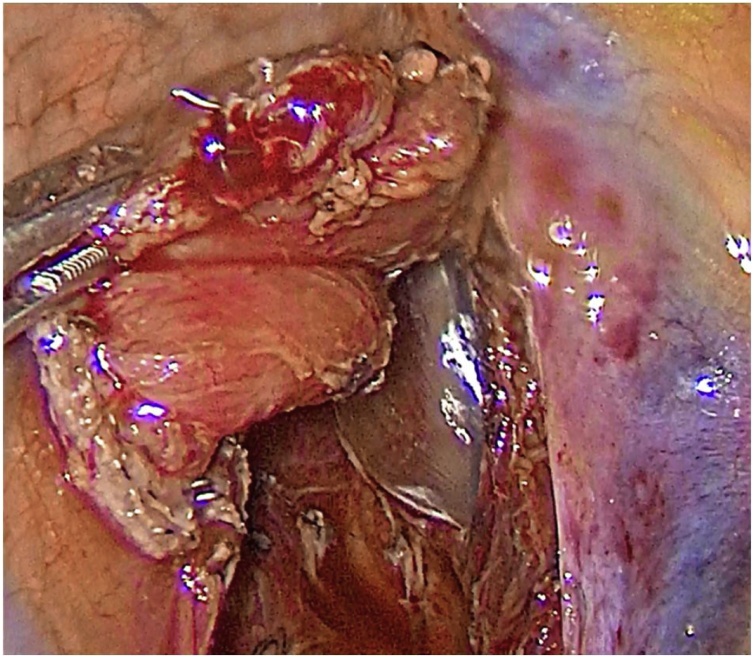
The tracheal cuff is seen in the trachea through the 30 mm rupture.

**Fig. 4:**
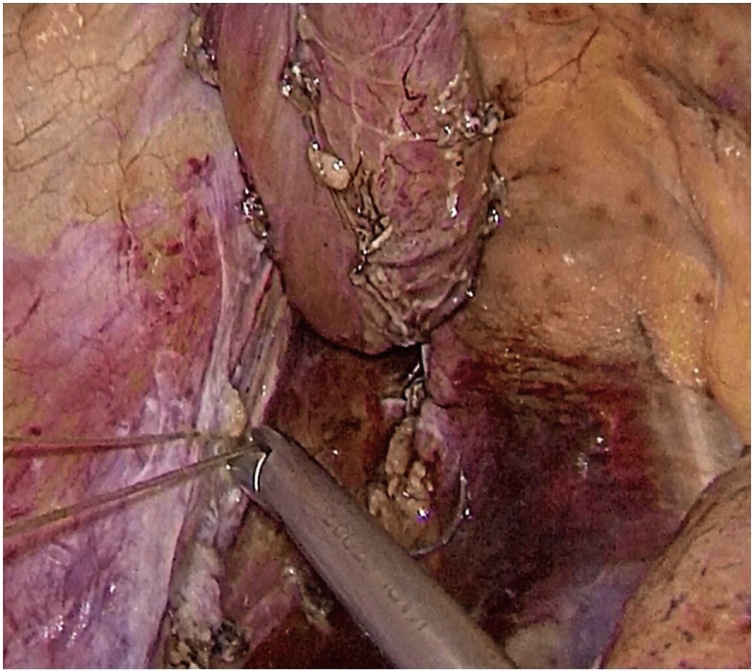
The ruptures are sewn with Stratafix 3.0 suture.

Next, the esophagus was cut off using the Endopath Echelon Flex 45 stapler (Ethicon, USA) with a 45 mm yellow cassette at the distance of 5 cm from the tumor. After the specimen was removed two defects of the tracheobronchial tree were detected. The first rupture with sizes of 15 mm was situated in the area of the left main bronchus right below the tracheal bifurcation (Fig. 2). Another defect was located on the membranous part of the trachea and had the size of 30 mm (Fig. 3). The intraoperative ruptures were sewn on an endotracheal tube using continuous Stratafix 3.0 suture (Fig. 4). In the area of tracheal rupture the suture was additionally covered by mediastinal pleura. An underwater air leak test was made for the impermeability of the suture.

**Fig. 5:**
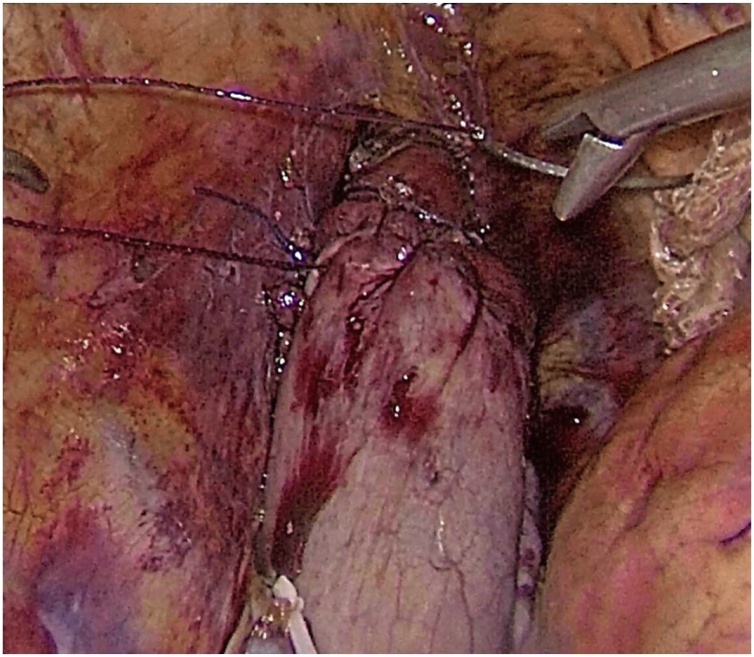
Thoracoscopic hand-sewn esophagogastroanastomosis.

Next, the resected part of the esophagus was placed into a plastic container and moved into the abdominal cavity through the esophageal opening of the diaphragm. Then the gastric tube was pulled into the mediastinum and trial comparison of the edges of the anastomosis without tension was performed. The gastric tube was fixed to the remaining part of the esophagus by Vicryl 2.0 suture. After that, the stomach and the esophagus were opened using electrocauter. The esophagogastroanastomosis was performed using V-loc 2.0 suture at the level of the pleural dome (Fig. 5). Then, the nasogastric tube was inserted below the anastomosis for decompression. In the next place, the anastomosis zone was covered with stomach using Vicryl 2.0 sutures with the purpose of antireflux protection.

A methylene blue test of the anastomosis was performed; there was no data for dehiscence and leakage. Then the pleural cavity was drained on the right side by two drain tubes; trocars were removed and trocar wounds were sutured. The resected specimen was removed from the abdominal cavity and sent to the laboratory for pathological examination.

**Fig. 6:**
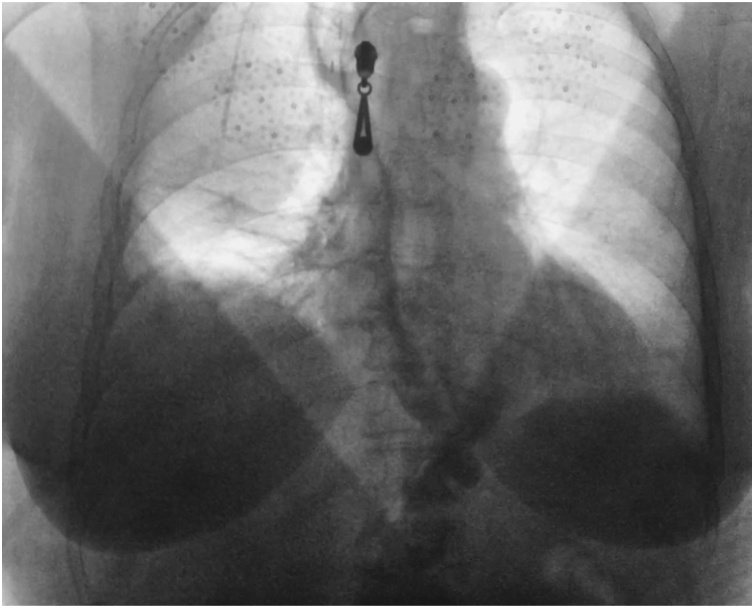
Postoperative esophagogram with Urografin swallow.

The surgery was performed by the team, consisted of 3 experienced in thoracoscopy surgeons. The overall operative time was 345 min. The patient was extubated 1 h after the end of the operation with no signs of respiratory disorders. There was no air leakage through the pleural drains and they were removed on 5th postoperative day. The woman tolerated the surgery well and started to take in a liquid food on the 6th postoperative day. On the 10th day after the surgery an esophagogram with liquid contrast (Urografin) was performed (Fig. 6).

On the 12th day after the surgery the patient was discharged from the hospital in a good state of health with standard dietary and medical recommendations. There were no postoperative complications, no symptoms of dysphagia, nausea or vomiting.

## 3 Discussion

The esophageal resection with right-side thoracoscopy and intrathoracic anastomosis of mediastinal esophagus and gastric tube has a number of advantages compared to thoracotomic access. They are less operative trauma, reduced blood loss, convenience in forming a gastroesophageal anastomosis, faster and better straightening of the right collapsed lung. The improvement of thoracoscopic technique leads to decrease of the operative time and makes it comparable to thoracotomic access operation duration. In comparison with transhiatal interventions, the advantage of this operation is the perfect exposure that allows performing complete esophageal dissection and possible en bloc resection [10,11].

A damage of the membranous part of the trachea and bronchi during esophagectomy is a rare complication which can be caused by the technical difficulties of dissecting the esophageal tumor of a large size, excessive traction of the esophagus in the area adjacent to the membranous part of the trachea, and is facilitated by sclerotic changes of muscle fibers connecting the esophagus and the trachea. The membranous part of the trachea has a delicate, fragile structure, which requires an adequate selection of suture material for its suturing. The use of intracorporeal continuous Stratafix 3.0 suture with subsequent strengthening by the mediastinal pleura makes it possible to achieve impermeability of the respiratory tract. If possible, early postoperative extubation of the patient contributes to the prevention of respiratory failure.

More thorough selection of patients undergoing thoracoscopic esophageal resection together with gentle manipulations with the esophagus in the area adjacent to the trachea can prevent intraoperative tracheobronchial damages. Timely diagnosis of such serious complications makes it possible to successfully manage them using thoracoscopic techniques.

## 4 Conclusion

We have successfully managed intraoperative tracheal and bronchial rupture during thoracoscopic resection for esophageal cancer with use of the intracorporeal suture.

## Conflict of interest

The authors declare no conflicts of interest. The authors have no financial, consultative, institutional and other relationships that might lead to bias or conflict of interest.

## Sources of funding

None.

## Ethical approval

Ethical approval has been exempted by our institution, NGHCI Railway Clinical Hospital at the “Rostov-Glavnyy” station, OAO Russian railways, Rostov-on-Don, Russian Federation.

## Consent

Written informed consent was obtained from the patient for publication of this case report and accompanying images. A copy of the written consent is available for review by the Editor-in-Chief of this journal on request.

## Author’s contribution

Khitaryan А. – conceptualization, funding acquisition, investigation, methodology, project administration, resources, supervision, verification, writing original draft, writing review & editing.

Miziev I. – conceptualization, funding acquisition, investigation, methodology, project administration, resources, supervision, verification, writing original draft, writing review & editing.

Veliev K. – conceptualization, writing original draft, writing review & editing.

Voronova O. – data curation, investigation, writing original draft, writing review & editing.

Golovina A. – conceptualization, formal analysis, methodology, project administration, data curation, formal analysis, software, resources, visualization, writing original draft, writing review & editing.

Zavgorodnyaya R. – data curation, investigation, writing original draft, writing review & editing.

Kovalev S. – data curation, investigation, writing original draft, writing review & editing.

Alibekov A. – data curation, investigation, writing original draft, writing review & editing.

Orekhov А. – conceptualization, writing original draft, writing review & editing.

## Registration of research studies

None.

This publication is neither ‘first-in-man study’ nor a research.

## Guarantor

Khitaryan А.G.

Golovina A.A.

## Provenance and peer review

Not commissioned, externally peer reviewed.
